# Helminth communities from two urban rat populations in Kuala Lumpur, Malaysia

**DOI:** 10.1186/1756-3305-5-47

**Published:** 2012-03-07

**Authors:** Siti N Mohd Zain, Jerzy M Behnke, John W Lewis

**Affiliations:** 1 School of Biological Sciences, University of Malaya, 50603 Kuala Lumpur, Malaysia; 2 School of Biology, University of Nottingham, University Park, Nottingham NG7 2RD, UK; 3 School of Biological Sciences, Royal Holloway, University of London, Egham, Surrey, TW20 0EX, UK

**Keywords:** *Rattus norvegicus*, *Rattus rattus*, helminths, nematodes, helminth species diversity, *Hymenolepis diminuta*, *Hymenolepis nana*, *Mastophorus muris*, *Nippostrongylus brasiliensis*, *Heterakis spumosum*

## Abstract

**Background:**

The prevalence of parasitic infections among commensal animals such as black and brown rats in many tropical countries is high and in comparison with studies on rodents in temperate climates, little is known about the community structure of their parasites. Rodent borne parasites pose threats to human health since people living in close proximity to rodent populations can be exposed to infection.

**Methods:**

The helminth community structures of two urban rat populations in Kuala Lumpur, Malaysia were investigated. The rats were from two contrasting sites in the city caught over a period of 21 months in 2000-2002.

**Results:**

Eleven species of helminth parasites comprising seven nematodes (*Heterakis spumosum, Mastophorus muris, Nippostrongylus brasiliensis, Syphacia muris, Pterygodermatites tani/whartoni, Gongylonema neoplasticum, Angiostrongylus malaysiensis*), three cestodes (*Hymenolepis *(*Rodentolepis*) *nana, H. diminuta *and *Taenia taeniaeformis*) and one acanthocephalan (*Moniliformis moniliformis*) were recovered from 346 *Rattus rattus *and 104 *R. norvegicus *from two urban sites, Bangsar and Chow Kit, during 2000-2002. *Rattus rattus *harboured over 60% of all helminths compared with *R. norvegicus*, although both host species played a dominant role in the different sites with, for example *R. norvegicus *at Bangsar and *R. rattus *at Chow Kit accounting for most of the nematodes. Overall 80% of rats carried at least one species of helminth, with the highest prevalences being shown by *H. diminuta *(35%), *H. spumosum *(29.8%) and *H. nana *(28.4%). Nevertheless, there were marked differences in prevalence rates between sites and hosts. The influence of extrinsic (year, season and site) and intrinsic (species, sex and age) factors affecting infracommunity structure (abundance and prevalence of infection) and measures of component community structure were analyzed.

**Conclusions:**

Since at least two species of rat borne helminths in Kuala Lumpur have the potential to infect humans, and these showed high prevalences in the rats, the assessment and regular monitoring of infections carried by wild rodents have important roles to play in public health.

## Background

The prevalence of parasitic infections among commensal animals in many tropical countries is high and poses threats to human health since people living in close proximity to rodent populations that act as reservoirs of infection, or to secondary hosts, can be exposed to infection. An extreme example of this is given by bubonic plague, the causative bacterium *Yersinia pestis *being transmitted by rat fleas, but there are many other examples of human diseases that have their origins in commensal rodent populations (e.g. Weil's disease, etc.) including helminths (e.g. trichinosis) [1-5]. Assessment and regular monitoring of infections carried by wild rodents therefore, have important roles to play in public health.

European and N. American wild rodents have been intensively investigated over the last decades with respect to their parasitic infections [6-12], but in comparison the parasite populations of wild rodents from the Far East have been poorly documented, and very little is known about their ecology and epidemiology. Several projects in Europe and the USA have focused on the factors that affect parasite communities including biogeography and abiotic parameters as well as host density and life history [9-14]. However, most studies of rodent parasites from the Far East are little more than species lists, and records of overall prevalence of species [15-27] with differing habitats and relatively small sample sizes [28]. To date there have been no comprehensive studies relating factors responsible for variation in parasite burdens of rodent communities in Malaysia nor any studying helminth communities over an extended period of time in this region.

The two dominant commensal rat species, the brown rat (*Rattus norvegicus*) and the black rat (*R. rattus*), are distributed worldwide [29]. Both rodents inhabit urban cities, being commonly found around dumpsites, around cross-pits, in sewer systems and storm drains [30] and both have been well-studied because of their medical and economic importance [2].

Therefore, the objectives of the present work were to investigate the diversity of helminth parasites in two species of commensal rats (*R. rattus *and *R. norvegicus*) from two urban sites in Kuala Lumpur. Additionally, the abundance of helminth species was monitored over a three year period, measures of component and infracommunity structure were calculated and interactions of helminth parasites with both intrinsic and extrinsic factors that are known to affect parasite abundance in other rodent species, were assessed. Of particular interest, was the relative role of these two rat species as hosts and reservoirs of the rodent helminth parasites endemic in the city, and particuarly those species that are transmissible to the human inhabitants of the city.

## Methods

### Study sites

Kuala Lumpur, the capital city of Malaysia is characterized by a tropical climate of high temperature and high humidity all year round with temperatures ranging between 30-36°C and with rainfall fairly even throughout most of the year but typically heavier during the monsoon season between October to February.

For the present work, the study sites were chosen based on the marked differences in the habitat and resources that they provide for rodents. Chow Kit (03°09' 53.75" N, 101°41' 56.84" E) is the largest wet market in Kuala Lumpur, a wet market being a fresh food market of a type commonly found in Asian countries. The name is derived from the extensive use of water in the markets in order to wash the floors, keep the fruits and vegetables fresh, and keep fish and shellfish alive. Traders sell an extensive range of raw food including fruits, vegetables, seafood and meat. Here, tons of rubbish are collected daily and deposited into several steel containers. Excess garbage falls to the ground forming temporary grounds for rats to forage in. In contrast, Bangsar (03°7' 50.78" N, 101°40' 19.05" E) is an affluent residential suburb on the outskirts of Kuala Lumpur with mixed residential sites. The hawker centres, restaurants and roadside stalls sell cooked food and rodents found here thrive on leftovers.

### Collection and examination of rats

Rodents were trapped regularly by the vector control unit of the Kuala Lumpur Municipality (DBKL) as part of their rodent control measure. Rats were caught from both vicinities over a span of 4 days and 3 nights for 21 months in 2000-2002. Steel wire traps were used and were baited with tapioca and dried fish. Rats were removed alive from these traps and killed with chloroform prior to post mortem examination. They were examined immediately afterwards and morphometric measurements were taken, together with records of fur colour, to assist in distinguishing between the two species. The lengths of head and body, tail, hind foot and ear were recorded and with body weight, these parameters enabled the establishment of age classes.

A complete post-mortem was undertaken on freshly killed specimens, the alimentary tract together with its offshoots being carefully scrutinized for helminth parasites. When found, these were removed carefully, identified, counted and preserved in 70% ethanol.

### Age classes of rats

Rats were allocated to three age classes on the basis of body weight, but since the two species differ markedly in adult weight, the ranges were different for each species. For *R. norvegicus *rats < 140 gm in weight were allocated to age class one, those 140 to < 240 gm into age class two, and age class three comprised rats > 240 gm. For *R. rattus *age class one comprised rats < 90 gm, age class two 90 to < 150, and age class three > 150 gm. The choice of the borderline between age class one and age class two for each species was based on MacDonald & Barrett [29]. The remaining rats were divided into two approximately equal groups numerically, with the lighter half being considered as young but sexually mature adults, and the heavier half as older animals.

### Seasonal cycles

The study was conducted across three calendar years (2000, 2001 and 2002), but local seasons did not fit conveniently within the January to December period. The dry season begins in March and extends to September, with the wet season occurring from October to February. Analyzing data with calendar year as a factor would have resulted in each year beginning and ending with a wet season, with the dry season in-between, and a split in the continuity of the wet season across the year divide. For this reason we fitted seasonal cycles rather than year as the more relevant factor, beginning with the dry season of seasonal cycle 1 and continued for 3 cycles, ending in the wet season of cycle 3.

### Data analysis

Prevalence data (percentage of animals infected) are shown with 95% confidence limits (95% CL, lower limit - upper limit), calculated as described previously [31], using bespoke software and were analyzed by maximum likelihood techniques based on log linear analysis of contingency tables in the software package SPSS 16. These analyses were confined to helminths that showed an overall prevalence greater than 10%. Initially, full factorial models incorporated host species (2 levels, *R. norvegicus *and *R. rattus*), seasonal cycle (3 levels, cycle 1, 2 and 3), site (2 levels, Chow Kit and Bangsar), season (2 levels, wet and dry season), sex (2 levels, males and females), age (3 age classes) and infection as a binary factor (present/absent). The backward selection procedure was implemented to derive minimum sufficient models for which the likelihood ratio of χ^2 ^was not significant. The importance of each term (i.e. interactions involving infection) in the final model was assessed by the probability that its exclusion would alter the model significantly.

Because the analysis was based on seven factors (host, site, seasonal cycle, season, age, sex and presence/absence of infection), full factorial models were complex and therefore when relevant, a two stage analysis was adopted. First we explored the full factorial model seeking evidence that there was a difference between hosts, and if there was, we explored this further. Secondly we fitted models for each rat species in turn and examined all relevant interactions in these.

Helminth species richness was analyzed by GLM (with normal errors) in SPSS in a two step procedure, first fitting models that incorporated all factors, main effects and only 2-way interactions, for each host species in turn. These were simplified by the backward selection procedure, with stepwise removal of non significant terms, eventually deriving a minimum sufficient model that retained only the significant interactions, main effects associated with those interactions whether significant or not, and any additional significant main effects. Then, we added host species as an additional factor (2 levels, *R. norvegicus *and *R. rattus*) with all relevant 2-way and 3-way interactions. These models were then simplified by the backward selection procedure until a minimum sufficient model was obtained, as above.

The degree of aggregation in the data was calculated by the index of discrepancy *(D *[32]) and the index of dispersion (*I*, variance to mean ratio). Frequency distributions of individual taxa were also tested for goodness of fit to negative binomial, positive binomial and Poisson models by Chi^2 ^[33].

For analysis of parasite abundance data, we first fitted generalized linear models with negative binomial errors in R version 2.2.1 (R Core Development Team), using the glm.nb routine in the MASS library of R [34]. All parametric models were checked for approximately normal or negative binomial distribution of residuals, and if the former did not conform to normal distribution, we transformed the raw data accordingly.

When neither of the above generated satisfactory models, we resorted to non-parametric tests, first examining each of the main effects in turn, either with the Mann-Whitney U test (2 levels) or the Kruskal-Wallis test (3 levels) on the combined data-set from both species of rats, and then on each of the rat species in turn.

## Results

### Hosts

A total of 486 rats were autopsied including, 140 *R. norvegicus *and 346 *R. rattus*. Four other species were also encountered and these were *R. argentiventus, R. annandalei, R. exulans *and *R. tiomanicus*. None of these species exceeded 7% of the catch at either site and we do not report further on these species of *Rattus *in the current work. Table 1 shows the numbers by host species, site and age class. For *R. norvegicus *the sex ratio was close to 1, with 52.9% males and 47.1% females. For *R. rattus*, more females were sampled (57.7% females and 42.3% males). One rat was not sexed and was excluded from all analyses in which sex was a consideration. More of each species were caught in the dry season (*R. norvegicus *60.7% and 39.3%, *R. rattus *55.45 and 44.6% in dry and wet seasons, respectively), which was longer than the wet season (7 months compared with 5). Fewer of each species were caught in 2000 and most in 2001 (*R. norvegicus *12.9%, 50.7% and 36.4%, *R. rattus *5.8%, 57.4% and 36.8.0% in 2000, 2001 and 2002, respectively). However, the analyses were conducted in relation to seasonal cycles that enabled actual seasons to be fitted chronologically, ranging from March of each year to the following February. In this arrangement for *R. norvegicus *the percentages of rats in seasonal cycles 1, 2 and 3 were 17.9, 53.6 and 28.6% and for *R. rattus*, 13.9, 60.3 and 25.8%, respectively. With six factors fitted (host species, season, cycle, site, sex and age), log-linear analysis revealed a complex model in which all factors played a significant role in 8 sets of interactions (five 3-way and three 2-way interactions, full model likelihood ratio χ^2^_100_= 72.53, *P *= 0.982).

**Table 1:** Numbers of rats examined by species, site and age class

		Age class				
				
Species	Site	Class 1	Class 2	Class 3	Total	Total
*R. norvegicus*	Chow Kit	15	33	12	60	140
	Bangsar	22	18	40	80	
*R. rattus*	Chow Kit	38	119	128	285	345
	Bangsar	11	23	26	60	
Total by species	*R. norvegicus*	37	51	52	140	485
	*R. rattus*	49	142	154	345	
Total by site	Chow Kit	53	152	140	345	485
	Bangsar	33	41	66	140	
Total		86	193	206		485

This table excludes one *R. rattus *that was not sexed

### Host age

**Figure 1:**
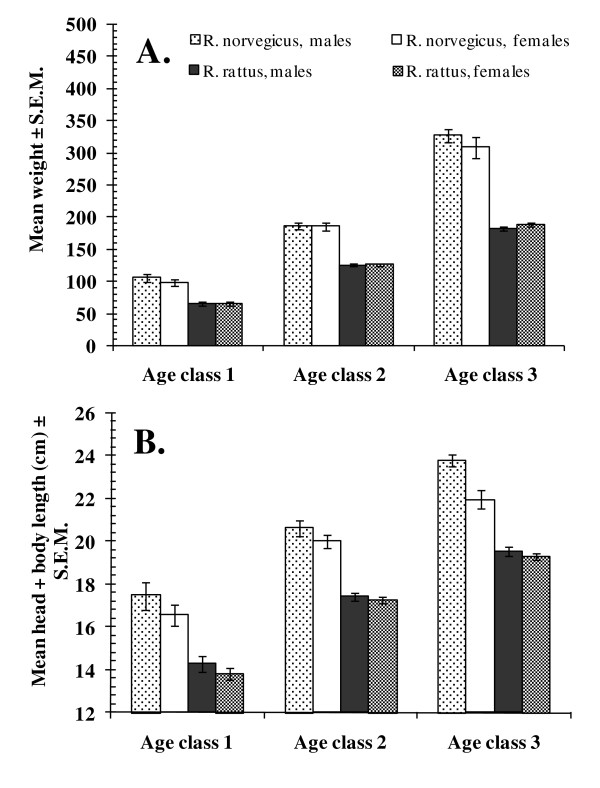
**Analysis of morphometric measures in relation to the age classes**. Rats were allocated to 3 age classes as described in the text. Body weight (A) where analysis was initially by 3-way GLM, but sex and its interactions were not significant and therefore was removed from the model. In a 2-way GLM with host species and age as factors, for main effect of host species *F*_1,479 _= 565.8, *P *< 0.001; for main effect of age *F*_2,479 _= 908.9, *P *< 0.001 and for the interaction *F*_2,479 _= 90.0, *P *< 0.001 and the model adjusted *R*^2 ^= 0.83; head and body length (B) where analysis is by 3-way GLM, with host species, sex and age as factors, and this gave for the main effects of host species *F*_1,477 _= 370.4, *P *< 0.001, age *F*_2,477 _= 337.5, *P *< 0.001, host sex *F*_1,477 _= 19.2, *P *< 0.001 and for the interaction between host species and sex *F*_1,479 _= 7.8, *P *= 0.005. No other interactions were significant with the model adjusted *R*^2 ^= 0.68.

In order to confirm that the three age classes reflected the growth and aging of rats, i.e. increased with host age from age class 1 through to age class 3, we first conducted a Principal Components Analysis, fitting host weight, head and body length, tail length, hind foot length and ear length as separate components. For *R. norvegicus *Principal Component 1 (PC1) accounted for 61.5% of the variance and showed the expected positive correlation with each parameter in turn, ranging from *R *= 0.92 for head + body length, to *R *= 0.442 for ear length. For *R. rattus *PC1 accounted for 52.0% of the variance and showed positive correlations with each parameter ranging from *R *= 0.90 for body weight to *R *= 0.39 for ear length and increased markedly across the three age classes. In *R. norvegicus*, but not *R. rattus*, there was also a significant difference between the sexes (*F*_1, 134 _= 4.54, *P *= 0.035; for males mean PC1 = 0.293 ± 0.114, and for females -0.329 ± 0.114). With the exception of ear length, each parameter had the lowest mean in age class 1 and increased through to age class 3. Ear length increased from age class 1 to age class 2 but no further (*F*_2,475 _= 21.8, *P *< 0.001). In all cases there was a significant difference between the host species, confirming that morphometric parameters were always higher in *R. norvegicus *compared with *R. rattus *(Figure 1). The rate of increase in body weight was higher across the three age classes in *R. norvegicus *compared with *R. rattus *(Figure 1A). For head plus body length there was a 2-way interaction between host species and sex arising from the body length being very similar in male and female *R. rattus*, but increasing more quickly in male compared with female *R. norvegicus *across the three age classes (Figure 1B). Hind foot length varied between the sexes (*F*_1,472 _= 33.6, *P *= 0.001), means being consistently higher among males of both species, but the discrepancy between the sexes was higher among *R. norvegicus *compared with *R. rattus *(2-way interaction between host species and sex, *F*_2,472 _= 39.6, *P *< 0.001). Tail length also varied between the sexes across both species (*F*_1,471 _= 11.3, *P *= 0.001) but contrary to the other measures tail length was generally longer in females (data not shown).

### Regional helminth community structure

The combined total of all helminths recovered in the survey was 13,800 with slightly more cestodes (51.0%) than nematodes (48.0%), and acanthocephala being poorly represented (1.0%). When the percentage distribution of helminths was calculated, *R. rattus *harboured almost two thirds of all helminths compared with *R. norvegicus*, and this was similar across all the higher taxa, except for larval cestodes which were only found in *R. rattus *(Table 2).

**Table 2:** Percentage distribution of higher taxa by host species and site

	Host							
		
	*R. norvegicus*		*R. rattus*		Hosts combined		Sites combined	
			
	Chow Kit	Bangsar	Chow Kit	Bangsar	Chow Kit	Bangsar	*R. norvegicus *	*R. rattus*
All helminths as a % of grand total*	4.3	13.7	72.6	9.4	76.9	23.1	18.0	82.0
All helminths adjusted for sampling effort**	10.9	26.3	38.9	24.0	49.8	50.3	37.2	62.9
Nematodes**	7.1	38.3	47.2	7.4	54.3	45.7	45.4	54.6
Adult cestodes**	13.5	17.7	33.5	35.2	47.0	52.9	31.2	68.7
Larval cestodes**	0	0	100	0	100	0	0	100
Acanthocephala**	17.4	12.3	10.8	59.5	28.2	71.8	29.7	70.3

* Values based on total number of helminths recovered from each site without adjustment for sample size

** Percentage distribution calculated with sample size taken into consideration

Eleven species of helminths were identified, comprising 3 species of cestodes, two of which were adult intestinal forms and one larval, seven species of nematodes and 1 acanthocephalan (Table 3).

**Table 3:** Prevalence (% of hosts infected) of helminth taxa by host species and site

		Species				
			
		*R. norvegicus*		*R. rattus*		
			
Taxon	Species	Chow Kit	Bangsar	Chow Kit	Bangsar	Combined
Nematodes	*Mastophorus muris*	21.7	3.8	22.8	8.3	17.7
	*Syphacia muris*	0	0	12.3	0	7.2
	*Nippostrongylus brasiliensis*	1.7	17.5	20.0	13.3	16.5
	*Gongylonema neoplasticum*	0	0	0.7	0	0.4
	*Pterygodermatites whartoni/tani*	1.7	3.8	4.6	5.0	4.1
	*Heterakis spumosum*	18.3	18.8	37.9	18.3	29.9
	*Angiostrongylus malaysiensis*	1.7	1.3	1.4	0	1.2
All nematodes		38.3	36.3	67.4	40.0	55.3
Cestodes	*Hymenolepis diminuta*	28.3	57.5	32.3	33.3	36.1
	*Hymenolepis nana*	21.7	21.3	29.8	36.7	28.2
	*Taenia taeniaeformis*	0	0	1.4	0	0.8
All adult cestodes		48.3	71.3	58.2	68.3	60.8
All larval cestodes		0	0	1.4	0	0.8
All cestodes		48.3	71.3	58.9	68.3	60.8
Acanthocephala	*Moniliformis moniliformis*	8.3	10.0	8.1	8.3	8.5
All helminths		66.7	81.3	82.5	80.0	80.0

### Measures of component community structure

The percentage distribution of helminths between the two sites was approximately equal when host sample size was taken into consideration, although in terms of actual worms recovered, more than three times as many came from the higher sample of rats from Chow Kit (Table 2). Over four times as many helminths were recovered from *R. rattus *compared with *R. norvegicus *in the raw data-set, but when we controlled for sample size the difference was almost two-fold (Table 2). Interestingly *R. norvegicus *trapped at Bangsar, and *R. rattus *at Chow Kit accounted for most of the nematodes, so the two host species each played a dominant role in one of the two sites in this context. About twice as many adult cestodes came from *R. rattus *compared with *R. norvegicus*, and this was consistent in both sites. However, all larval cestodes (Table 2) came from *R. rattus *in Chow Kit, and the majority of acanthocepahalans from *R. rattus *in Bangsar.

Of the eleven species of helminths identified (Table 3), the majority were shared by both rat species in both sites. All the species were recovered from *R. rattus *in Chow Kit, although four species (*S. muris*, *G. neoplasticum*, *T. taeniaeformis *and *A. malaysiensis*) were absent from Bangsar. The first three of these were not found in *R. norvegicus *at either site.

Overall, 80.0% of rats harboured at least one helminth species (Table 3) with high prevalence values being shown by *H. diminuta *(36.1%), *H. spumosum *(29.9%) and *H. nana *(28.2%). Nevertheless, there were marked differences in prevalence between sites and hosts.

Simpson's index of diversity was highest in *R. rattus *from Chow Kit, and lowest in *R. rattus *from Bangsar (Table 4). The overall value for Simpson's index of diversity was 0.740, which is almost identical to that for *R. rattus *at Chow Kit. Combining the data for both sites, the value of Simpson's was very similar for both species (*R. rattus *= 0.727 and *R. norvegicus *= 0.729), and combining rat species it was not markedly different between sites (Chow Kit = 0.744 and Bangsar = 0.697).

**Table 4:** Measures of component community structure by host species and site

	Species			
	
	*R. norvegicus*		*R. rattus*	
	
	Chow Kit	Bangsar	Chow Kit	Bangsar
Total no. of helminth species identified	8	8	11	7
Berger-Parker dominance index	0.499	0.523	0.401	0.700
Dominant species	*H. nana*	*N. brasiliensis*	*H. nana*	*H. nana*
Simpson's index of diversity	0.676	0.652	0.741	0.487

The Berger-Parker dominance index was highest in *R. rattus *from Bangsar, where the dominant species was *H. nana *(Table 4). The remaining three combinations had very similar but lower Berger-Parker dominance indices, and in two of these *H. nana *was also the dominant species. However, in *R. norvegicus *from Bangsar *N. brasiliensis *was the dominant species.

### Measures of infracommunity structure

#### Mean species richness

**Figure 2:**
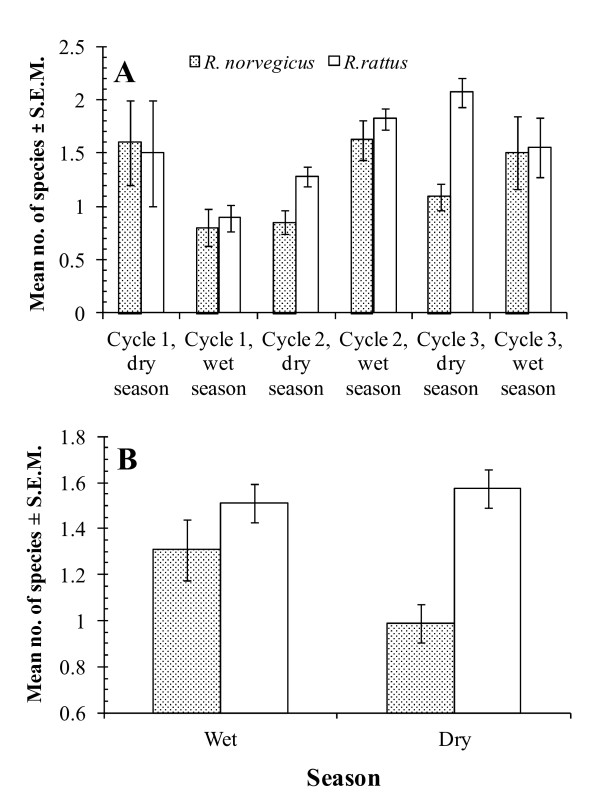
**Variation in helminth species richness**. Cyclical and seasonal variation in both hosts (A) and seasonal variation between hosts (B).

The overall mean number of helminth species harboured per host (all rats combined, n = 485) was 1.4 ± 0.05 (variance to mean ratio = 0.77). Analysis of helminth species richness restricted to *R. norvegicus *revealed only one significant 2-way interaction, seasonal cycle*season (*F*_2, 134 _= 6.1, *P *= 0.003). Mean species richness was higher in 2 wet seasons (cycles 2 and 3), but lower in the wet season of cycle 1 compared with the preceding dry season (Figure 2A).

For *R. rattus *a similar 2-way interaction was highly significant (seasonal cycle*season, *F*_2, 336 _= 7.57, *P *= 0.001) and the pattern of change was much the same in seasonal cycles 1 and 2, but not in cycle 3, when mean helminth species richness was higher in the dry season compared to the wet season much as in cycle 1 (Figure 2A). There were also two marginally significant main effects in *R. rattus*; mean helminth species richness was lower at Bangsar compared with Chow Kit (Table 5, *F*_1, 336 _= 4.03, *P *= 0.046), and increased with age, but then leveled off in age class 3 (*F*_1, 336_= 3.23, *P *= 0.041, values in age classes 1-3, = 1.18 ± 0.145, 1.69 ± 0.092, 1.53 ± 0.087, respectively). Neither the effect of site nor host age proved significant in the case of *R. norvegicus*. With respect to site, the trend was in the opposite direction to that in *R. rattus *although less marked (Table 5), and similarly in relation to age, the increase with age was delayed relative to *R. rattus *(mean helminth species richness in age classes 1-3, = 1.03 ± 0.142, 1.06 ± 0.123, 1.23 ± 0.118, respectively).

**Table 5:** Measures of infracommunity structure by host species and site

	Species			
	
	*R. norvegicus*		*R. rattus*	
	
	Chow Kit	Bangsar	Chow Kit	Bangsar
Mean number of species ± S.E.M.	0.950 ± 0.110	1.238 ± 0.096	1.633 ± 0.067	1.150 ± 0.100
Maximum number of species	3	3	5	3
Mean number of helminths ± S.E.M.	9.8 ± 2.29	23.7 ± 5.60	35.0 ± 4.3	21.6 ± 5.37
Mean number of nematodes ± S.E.M.	2.7 ± 0.96	14.6 ± 4.83	17.9 ± 3.45	2.8 ± 0.75
Mean number of cestodes ± S.E.M.	6.8 ± 2.14	9.0 ± 2.22	16.9 ± 2.43	17.8 ± 4.94
Mean Brillouin's index ± S.E.M.	0.127 ± 0.031	0.166 ± 0.029	0.247 ± 0.016	0.147 ± 0.031
Maximum Brillouin's index	0.850	0.930	1.170	1.050

To determine whether helminth species richness varied significantly between hosts, the significant effects from each model were then re-analyzed in a restricted model, incorporating host species as an additional factor, and fitting all interaction terms. The 2-way cycle*season interaction emerged the strongest (*F*_2, 473_= 12.57, *P *< 0.001, Figure 2A) but the seasonal effect differed significantly between hosts (2-way interaction host*season, *F*_1, 473_= 5.98, *P *= 0.015), because when the cycles were combined there was still a marked seasonal difference in the mean helminth species richess in *R. norvegicus*, but not in *R. rattus *(Figure 2B). In particular, mean species richness was high in *R. rattus *compared with *R. norvegicus *during the dry seasons of cycles 2 and 3 (Figure 2A).

There was also a significant difference between the rat species (main effect of host, *F*_1, 473_= 6.44, *P *= 0.011, *R. norvegicus *= 1.11 ± 0.073, *R. rattus *= 1.55 ± 0.059) and a weaker overall effect of age with hosts combined (main effect of age, *F*_2, 473_= 3.58, *P *= 0.029, mean helminth species richness in age classes 1-3, = 1.12 ± 0.103, 1.52 ± 0.078, 1.46 ± 0.072, respectively). Not surprisingly, there was a significant difference between the rat species in their mean helminth species richness values from the two sites (2-way interaction host*site, *F*_1, 473_= 4.62, *P *= 0.032, Table 5). In *R. norvegicus *the values were very similar in both sites but they were significantly higher in *R. rattus *at Chow Kit.

#### Measure of infracommunity diversity

The maximum number of helminth species ranged from 5 in *R. rattus *at Chow Kit to 3 in *R. rattus *at Bangsar and in *R. norvegicus *at both sites (Table 5).

**Figure 3:**
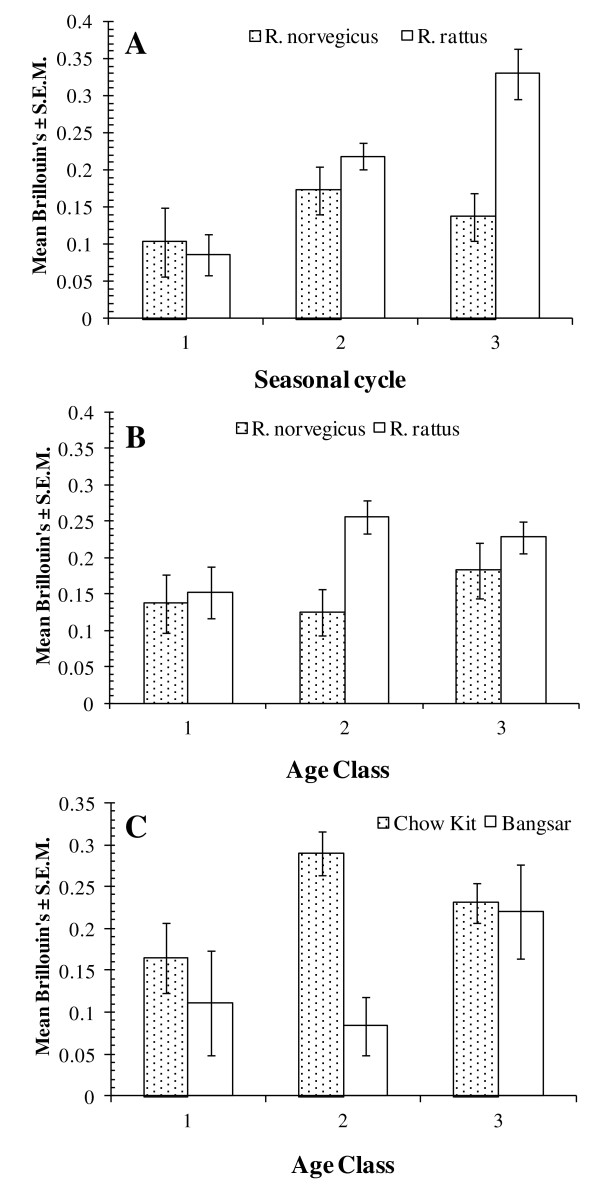
**Factors affecting Brillouin's index of helminth diversity in each rat species**. The effects of seasonal cycle (A), age class(B) in both host species and age class in *R. rattus *by site (C).

The analysis of Brillouin's index of diversity was complicated because parametric models did not converge. Therefore we used non-parametric tests to evaluate the effects of each of the factors in turn. All significant differences were clearly driven by values derived from *R. rattus*, since analyses that were confined to *R. norvegicus *did not reveal significant differences between levels within the specified factors. There was a highly significant difference between the host species (Mann-Whitney *U *test, *z*= -3.46, *P *= 0.001) with the mean value of Brillouin's being higher in *R. rattus *(0.23 ± 0.015) compared with *R. norvegicus *(0.15 ± 0.021). The mean value was highest in *R. rattus *from Chow Kit, because at Bangsar, the mean Brillouin's for *R. rattus *was much the same as for *R. norvegicus *at both sites (Table 5). The mean Brillouin's index increased significantly in *R. rattus *with each successive cycle (Kruskal-Wallis test χ^2^_2 _= 25.1, *P *< 0.001) but remained fairly stable in *R. norvegicus *(χ^2^_2 _= 1.2, *P *= NS; Figure 3a). Moreover, it was higher in age class 2 and 3 animals (Kruskal-Wallis test χ^2^_2 _= 6.4, *P *= 0.041; Figure 3b), compared with age class 1 in *R. rattus*, but showed no significant difference (χ^2^_2 _= 1.7, *P *= NS) between age classes in *R. norvegicus*. However, the pattern in *R. rattus *was predominantly that observed at Chow Kit (Figure 3c). At Bangsar, the Brillouin's index of diversity in *R. rattus *was still low in age class 2 rats, but increased in age class 3 rats to about the same value as in rats (*R. rattus*) from Chow Kit.

#### Prevalence of infection with individual helminth species

##### Hymenolepis diminuta

**Figure 4:**
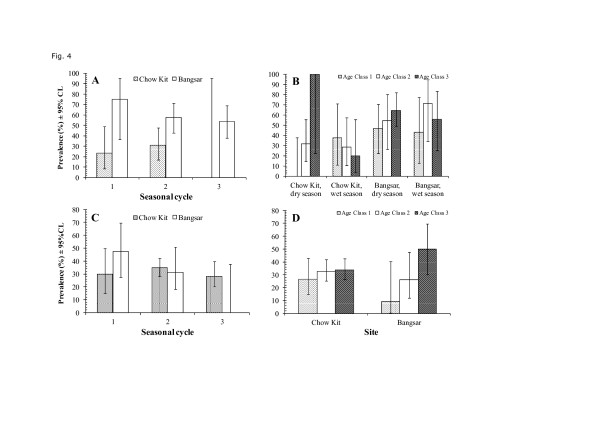
**Factors affecting prevalence of *Hymenolepis diminuta***. Variation between the two sites and across 3 seasonal cycles in *R. norvegicus *(A), in prevalence in the 3 age classes, two sites and two seasons, in *R. norvegicus *(B), in prevalence across the three seasonal cycles and in both sites in *R.rattus (C) *and in prevalence between age classes in both sites in *R. rattus *(D). See text for statistical significance of individual factors and interactions; for the fit of the overall minimal sufficient model incorporating both hosts *χ*^2^_183 _ =93.8, *P *= 1.0; for that restricted to *R. norvegicus χ*^2^_103 _ =70.9, *P *= 0.99, and for that restricted to *R. rattus χ*^2^_108 _ =66.6, *P *= 0.99.

This species was the most frequently occurring helminth with an overall prevalence of 36.1% [CL: 32.7-41.4]. Prevalence was similar in *R. rattus *in both sites and in *R. norvegicus *at Chow Kit, but almost twice as high at Bangsar in *R. norvegicus *(Table 3; for host*site*presence/absence of *H. diminuta *interaction, *χ*^2^_1 _= 6.5, *P *= 0.01). This higher prevalence in *R. norvegicus *at Bangsar compared with Chow Kit was evident in all three seasonal cycles (Figure 4A).

In the second phase of analysis we fitted models for each host species in turn. In both cases there was a significant seasonal cycle * season * presence/absence of *H. diminuta *interaction (For *R. norvegicus χ*^2^_2 _= 7.5, *P *= 0.024, *R. rattus χ*^2^_2 _= 7.43, *P *= 0.024). Prevalence was high in both rat species in the dry season of seasonal cycle 1, and then declined in the wet season, but thereafter prevalence values increased consistently with each successive season in *R. norvegicus*, whilst fluctuating at a lower level in *R. rattus *(data not illustrated).

In *R. norvegicus*, the age related prevalence of *H. diminuta *varied between both sites and seasons (analysis restricted to *R. norvegicus*, season*site*age*presence/absence *H. diminuta*, *χ*^2^_2 _= 6.3, *P *= 0.042). In the dry season at both sites there was a pattern of increasing prevalence with age, but in the wet season at Chow Kit prevalence declined with age and at Bangsar prevalence peaked in mature and not the oldest rats (Figure 4B).

For *R. rattus *the second significant term incorporating the presence/absence of *H. diminuta *was the seasonal cycle*site*presence/absence interaction (*χ*^2^_2 _= 8.4, *P *= 0.015). This arose because at Chow Kit prevalence was consistent across the 3 seasonal cycles, but fell from 48% to zero at Bangsar (Figure 4C).

The final interaction involving *H. diminuta *in *R. rattus *was site*age*presence/absence (*χ*^2^_2 _= 7.0, *P *= 0.030). At Chow Kit all three age classes of *R. rattus *showed similar prevalences whereas at Bangsar prevalence increased markedly from juveniles to the oldest individuals (Figure 4D).

##### Heterakis spumosum

This was the second most frequently occurring helminth with a prevalence of 29.9% [CL:26.0-34.2]. With the exception of *R. rattus *at Chow Kit, prevalence was remarkably uniform in *R. norvegicus *in both sites and in *R. rattus *at Bangsar but twice as high at Chow Kit in *R. rattus *(Table 3). Nevertheless, with both hosts combined there was a highly significant difference in the prevalence of *H. spumosum *between sites (analysis incorporating both rat species, site*presence/absence of *H. spumosum*, *χ*^2^_1 _= 8.1, *P *= 0.005; at Chow Kit prevalence was 34.4% (CL: 29.19-39.99%) and at Bangsar 18.6% (CL:12.95-25.79%)).

Although the overall prevalence was higher in *R. rattus *(34.5%) compared with *R. norvegicus *(18.6%), this difference was confounded by some interactions with age and season. In the dry season the prevalence was higher in all age classes in *R. rattus*, whereas prevalence in *R. norvegicus *was higher in age class 3 in the wet season (season*age*host*presence/absence of *H. spumosum*, *χ*^2^_2 _= 10.0, *P *= 0.007). When the effect of season was collapsed, the difference between age classes was far more pronounced in *R. norvegicus *(prevalence for age class 1 to 3 = 8.1%, 11.8% and 32.7%, respectively) compared with *R. rattus *(30.6%, 31.7% and 38.3%, respectively). Prevalence in both hosts declined in the wet season in seasonal cycle 1, and this drop was still evident in the dry season of seasonal cycle 2. Prevalence values then recovered in both hosts but dropped again in *R. rattus *during the wet season at the end of seasonal cycle 3 (season*cycle*host species*presence/absence of *H. spumosum*, *χ*^2^_2 _= 9.1, *P *= 0.011, Data not illustrated).

##### Hymenolepis nana

This was the third most frequently occurring species with an overall prevalence of 28.2% [CL:25.1-33.2]), and prevalence was higher in *R. rattus *(31.0%, [CL:27.3-37.1]) compared with *R. norvegicus *(21.4%, [CL:15.9-29.6]). This difference between hosts was consistent in both sites (Table 3), but was not found to be significant because prevalence was confounded by combinations of the other factors in the analysis. A model that included host species as a factor comprised 13 terms of which six 4-way interactions included presence/absence of *H. nana *together with various combinations of site, age, season and cycle. Sex did not play a role in determining the prevalence of *H. nana *(host species combined, males = 30.5% [CL:25.3-37.3]; females 26.4% [CL:22.0-32.6]).

In *R. norvegicus *prevalence varied across the three age classes and across the three seasonal cycles (age*season*cycle*presence/absence of *H. nana*, χ^2^_4 _= 9.5, *P *= 0.05). In the dry season prevalence was low in all three cycles in age class 3 rats and in the wet season it was higher in each case. In *R. rattus*, the only interaction involving the presence/absence of *H. nana *was one incorporating site and season in relation to age (site*season*age*presence/absence of *H. nana*, χ^2^_2 _= 15.6, *P *= 0.0004). There was little difference in prevalence between age classes 1 and 2, but in three data subsets the prevalence was lower in the age class 3 rats (Chow Kit dry and wet season and Bangsar in the wet season), the exception being Bangsar in the dry season.

##### Mastophorus muris

This nematode was present in 17.7% [CL:13.0-23.6] of rats, and almost twice as frequently encountered in *R. rattus *(20.3% [CL:16.0-25.2]) compared with *R. norvegicus *(11.4% [CL:7.0-17.7]).

The overall prevalence was up to 5 times higher at Chow Kit compared to Bangsar, and the higher prevalence at Chow Kit was common to both rat species (Table 3). Prevalence was also higher in females (21.5% [CL:17.6-25.9]) compared with males (13.2% [CL:10.3-16.7]) but not significant because of the confounding influences of more complex interactions with site, season and host. When the analysis was confined to *R. norvegicus*, only one of the 4 interactions included the presence/absence of *M. muris *(site*sex*age*presence/absence of *M. muris*, χ^2^_2 _= 7.5, *P *= 0.024) but no clear patterns were evident.

**Figure 5:**
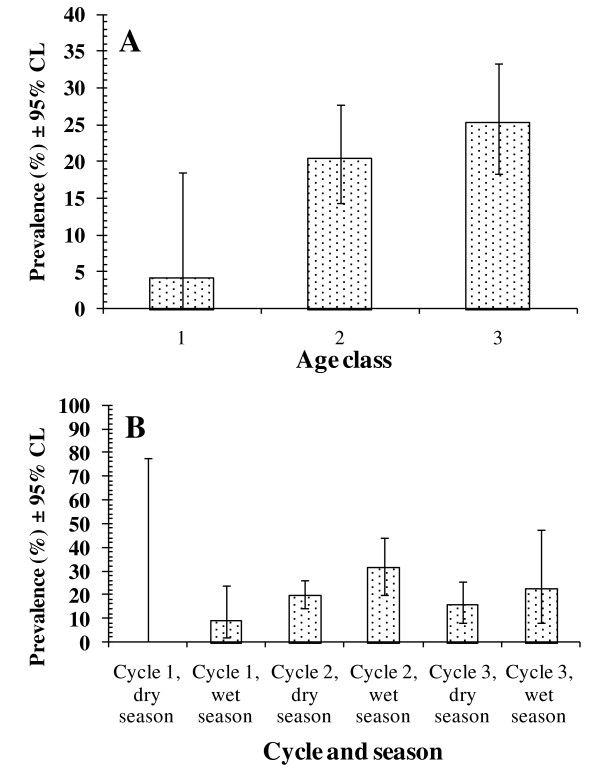
**Factors affecting the prevalence of *Mastophorus muris *in *R. rattus***. Effect of host age (A) and seasonal cycles (B). The overall model fit of the minimum sufficient model confined to *R. rattus *was *χ*^2^_121 _ =76.8, *P *= 0.99. See text for significance of individual factors and their interactions.

Analysis of *R. rattus*, however, revealed a very distinct picture with six interaction terms, four of which were 2 way interactions. There was a marked age effect (age*presence/absence of *M. muris*, χ^2^_2 _= 13.7, *P *= 0.0013) with an increasing prevalence of infection with age (Figure 5A). Prevalence varied significantly between sites (site*presence/absence of *M. muris, χ*^2^_1 _= 5.4, *P *= 0.02), being higher at Chow Kit as shown in Table 3, and between seasonal cycles, being highest in season cycle 2 (24.5%, [CL:20.9-28.6]), compared with cycle 1 (8.3% [CL:1.3-24.1]) and cycle 3 (16.9% [CL:8.8-28.8]; χ^2^_2 _= 7.8, *P *= 0.02). Finally, prevalence was also significantly higher in the wet compared with dry season (χ^2^_1 _= 4.0, *P *= 0.044; wet season = 23.4% [CL:16.7-31.4]; dry season = 17.8% [CL:11.3-26.4]). The increase from dry to wet season is apparent in each seasonal cycle, despite the differences in prevalence between cycles (Figure 5B).

##### Nippostrongylus brasiliensis

*Nippostrongylus brasiliensis *had an overall prevalence of 16.5% (95% [CL: 11.8-22.3]. Prevalence was higher in *R. rattus *compared to *R. norvegicus*, being 18.8% [CL:14.7-23.7] and 10.7% [CL:6.4-17.0] respectively, but with hosts combined prevalence values were similar at both sites (Chow Kit = 16.8% [CL:12.9-21.5]; Bangsar = 15.7% [CL:10.5-22.5]). However, when broken down by host and site (Table 3), prevalence can be seen to have been particularly low in *R. norvegicus *at Chow Kit. Statistically the difference between hosts was confounded additionally by season (model incorporating both hosts, host*site*season* presence/absence of *N. brasiliensis *χ^2^_1 _= 7.7, *P *= 0.0056 and model restricted to *R. norvegicus*, site*season*presence/absence of *N. brasiliensis*, χ^2^_1 _= 4.7, *P *= 0.03). Prevalence was higher in *R. rattus *(relative to *R. norvegicus*) in Chow Kit and at Bangsar in the dry seasons, but not in the wet season at Bangsar, when prevalence among *R. norvegicus *exceeded 40% (Data not shown).

**Figure 6:**
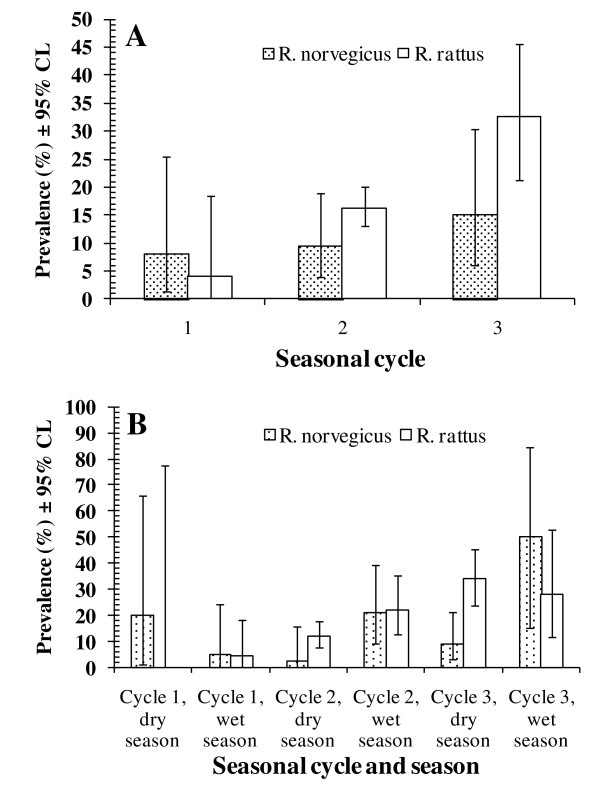
**Factors affecting variation in the prevalence of *Nippostrongylus brasiliensis***. The effect of 3 seasonal cycles (A) and seasonal cycles combined with both seasons in each cycle (B). The overall model fit of the minimum sufficient model incorporating both hosts was *χ*^2^_213 _ =113.9, *P *= 1.0, confined to *R. norvegicus χ*^2^_100 _ =43.4, *P *= 1.0 and confined to *R. rattus χ*^2^_108 _ =81.7, *P *= 0.97. See text for significance of individual factors and their interactions.

Statistical analysis revealed that the strongest effect was that arising from differences in prevalence between the seasonal cycles. This was evident in both hosts (cycle*presence/absence of *N. brasiliensis*, χ^2^_2 _= 23.2, *P *< 0.0001), in only *R. rattus *(χ^2^_2 _= 18.1, *P *< 0.0001), but not when *R. norvegicus *were analyzed alone. This seasonal cycle effect is shown for both species separately in Figure 6A. In both, prevalence increased with successive seasonal cycles, but the increase was more marked in *R. rattus*, *R. norvegicus *following a similar pattern but more slowly. When the season effect was added to that of seasonal cycles, an increasing prevalence in *R. rattus *was shown starting from the dry season of cycle 1 through to the dry season of cycle 3, and a similar but delayed pattern of change in *R. norvegicus *with a low in the dry season of cycle 2 (Figure 6B).

#### Measures of dispersion

The majority of helminths in the two rat species from both sites showed overdispersed (aggregated) distributions where *D *exceeded 0.7 and was generally much higher, and *I *exceeded 1 in all cases ranging from 3 to 146.8. The negative binomial constants *k*, were in general consistent with a negative binomial distribution. In 18 of the 30 subsets of data the distributions were not significantly different from those predicted by the negative binomial distribution and in 5 cases it was not possible to test for goodness of fit.

#### Abundance of infection with helminth species

The mean helminth burdens (all species combined) varied significantly between hosts (Table 5; χ^2^_1 _= 13.2, *P *= 0.0003), being almost twice as high in *R. rattus *(*R. norvegicus *= 17.7 ± 3.39; *R. rattus *= 32.7 ± 3.68), but not between sites, although arithmetically mean total helminth burdens were higher at Chow Kit (host species combined, Chow Kit = 30.6 ± 3.61; Bangsar = 22.9 ± 2.85). Nevertheless, there was also a significant difference between sites in *R. norvegicus *when the analysis was restricted to this species (Table 5; χ^2^_1 _= 9.13, *P *= 0.0025).

**Figure 7:**
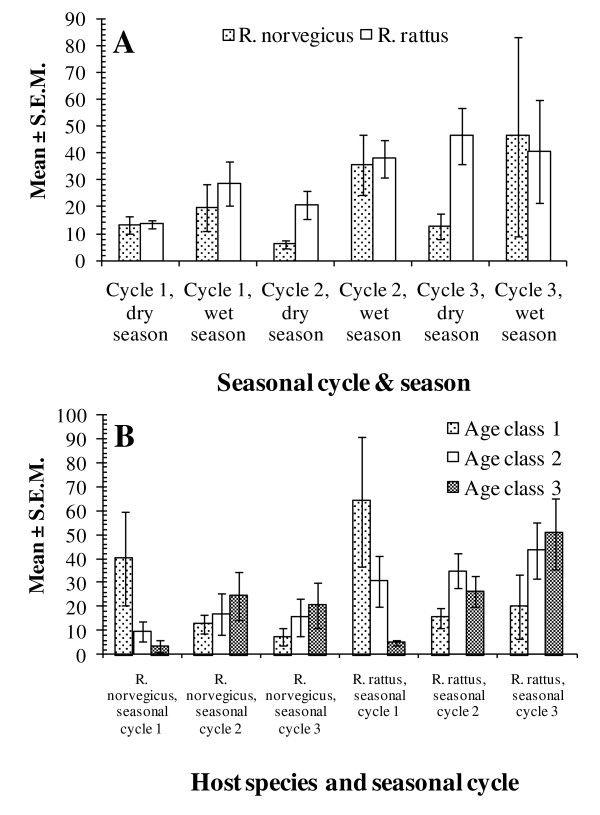
**Factors affecting the abundance of helminth species in both rat host species**. The effect of seasonal cycles and successive seasons on total helminth burdens (A) and variation in total helminth burden between age classes across 3 seasonal cycles (B). See text for statistical analysis.

The strongest influences on total helminth burden were season (χ^2^_1 _= 24.8, *P *< 0.0001) and seasonal cycle (χ^2^_2 _= 13.9, *P *= 0.0009), and the marked increase in helminth burdens from the dry to the following wet season was evident in both species (Figure 7A; for *R. norvegicus *χ^2^_1 _= 20.4, *P *< 0.0001; for *R. rattus *χ^2^_1 _= 8.2, *P *= 0.0043), except for *R. rattus *in cycle 3. There was also a steady increase in total helminth burdens with each successive seasonal cycle (hosts and sites combined, values for seasonal cycles 1, 2 & 3, were: 24.7 ± 5.74, 25.5 ± 3.38 and 36.7 ± 6.75, respectively).

There was a pronounced effect of host sex (χ^2^_1 _= 15.4, *P *= 0.0001), with male rats harbouring on average more worms than females (males = 34.9 ± 5.13; females = 22.9 ± 2.85). This effect of host sex was in the same direction in both rat hosts (*R. norvegicus *males = 23.0 ± 5.99, females = 11.9 ± 2.44; *R. rattus *males = 41.0 ± 7.07, females 26.6 ± 3.68) but, with other factors taken into account, was only significant in *R. rattus *(χ^2^_1 _= 9.15, *P *= 0.0025)

A highly significant 2-way interaction was found between host age and seasonal cycle on helminth abundance (χ^2^_4 _= 22.1, *P *= 0.0002). As mean helminth burdens accumulated with increasing age in both hosts in seasonal cycles 2 and 3, but not in seasonal cycle 1 when worm burdens clearly fell with increasing age (Figure 7B).

##### Hymenolepis diminuta

There was no significant difference in the overall abundance of *H. diminuta *between sites (hosts combined, Chow Kit = 2.7 ± 0.65; Bangsar = 3.6 ± 0.63) nor between hosts (sites combined, *R. norvegicus *= 3.2 ± 0.61; *R. rattus *= 2.9 ± 0.66), when other factors were taken into account. However, in *R. norvegicus *abundance was significantly higher at Bangsar (Table 6; χ^2^_1 _= 7.20, *P *= 0.0007).

**Table 6:** Abundance of helminth tax by host species and site

		Species				
			
		*R. norvegicus*		*R. rattus*		
			
Taxon	Species	Chow Kit	Bangsar	Chow Kit	Bangsar	Combined
Nematodes	*Mastophorus muris*	1.0 ± 0.36	0.2 ± 0.15	1.2 ± 0.18	0.2 ± 0.12	0.9 ± 0.12
	*Syphacia muris*	0	0	5.7 ± 2.83	0	3.4 ± 1.66
	*Nippostrongylus brasiliensis*	0.05 ± 0.05	12.4 ± 4.77	8.6 ± 1.80	1.4 ± 0.64	7.3 ± 1.33
	*Gongylonema neoplasticum*	0	0	0.007 ± 0.005	0	0.004 ± 0.005
	*Pterygodermatites whartoni/tani*	0.05 ± 0.05	1.1 ± 0.82	0.4 ± 0.17	0.5 ± 0.35	0.5 ± 0.18
	*Heterakis spumosum*	1.6 ± 0.88	0.9 ± 0.25	2.0 ± 0.30	0.7 ± 0.27	1.6 ± 0.21
	*Angiostrongylus malaysiensis*	0.03 ± 0.03	0.01 ± 0.01	0.04 ± 0.03	0	0.03 ± 0.02
All nematodes		2.7 ± 0.96	14.6 ± 4.83	17.9 ± 3.46	2.8 ± 0.75	13.6 ± 2.20
Cestodes	*Hymenolepis diminuta*	1.9 ± 0.65	4.2 ± 0.94	2.9 ± 0.78	2.7 ± 0.75	3.0 ± 0.50
	*Hymenolepis nana*	4.9 ± 2.10	4.7 ± 2.12	14.0 ± 2.36	15.1 ± 5.02	11.5 ± 1.59
	*Taenia taeniaeformis*	0	0	0.02 ± 0.01	0	0.01 ± 0.005
All adult cestodes		6.8 ± 2.14	9.0 ± 2.22	16.9 ± 2.44	17.8 ± 4.94	14.4 ± 1.63
All cestodes		6.8 ± 2.14	9.0 ± 2.22	16.9 ± 2.44	17.8 ± 4.94	14.4 ± 1.63
Acanthocephala	*Moniliformis moniliformis*	0.3 ± 0.14	0.2 ± 0.08	0.2 ± 0.04	1.0 ± 0.80	0.3 ± 0.11
All helminths		9.8 ± 2.29	23.7 ± 5.60	35.0 ± 4.30	21.6 ± 5.37	28.4 ± 2.81

**Figure 8:**
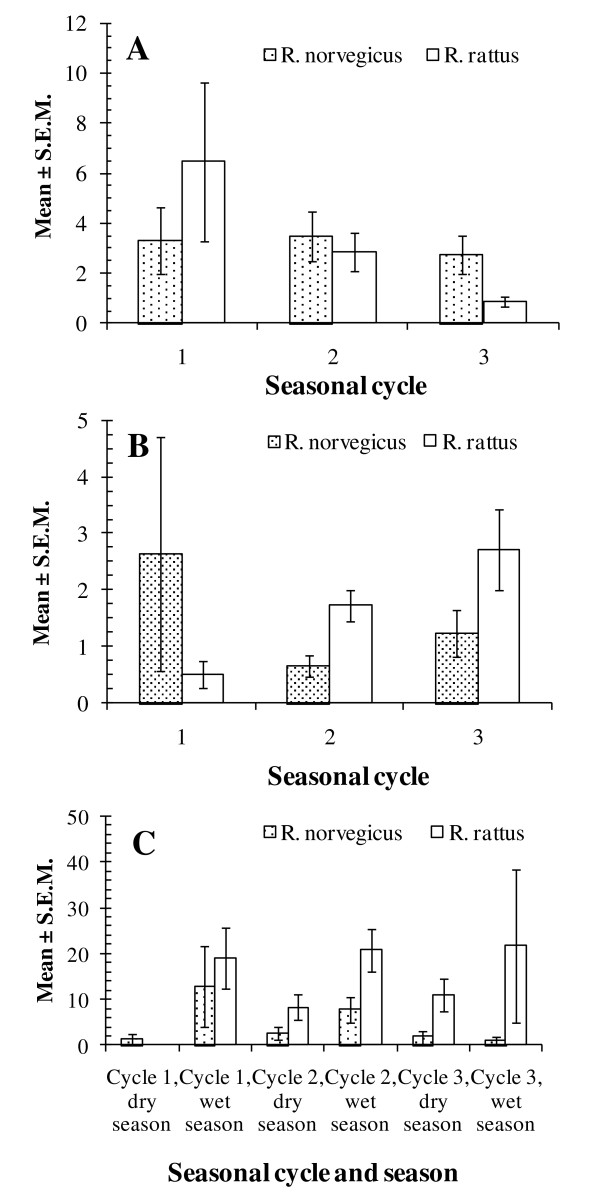
**Factors affecting the abundance of *H. diminuta*, *H. spumosum *and *H. nana *in both rat species**. The effect of seasonal cycles on the abundance of *H. diminuta *(A), *H. spumosum *(B) and also successive seasons and seasonal cycles on the abundance of *H. nana *(C). See text for statistical analysis.

In *R. rattus *abundance of *H. diminut*a clearly declined significantly across the 3 seasonal cycles (χ^2^_2 _= 11.26, *P *= 0.0036), whilst in *R. norvegicus *abundance was relatively stable (Figure 8A). There was no significant effect of age in either host, but there was a sex effect in *R. rattus *(χ^2^_1 _= 7.4, *P *= 0.0064), with females harbouring more worms than males (*R. rattus*, males 1.6 ± 0.29, females = 3.8 ± 1.11). In *R. norvegicus *abundance of *H. diminuta *was in the opposite direction but this was not significant when other factors were taken into consideration (males = 3.9 ± 1.02, females = 2.5 ± 0.61)

##### Heterakis spumosum

There was no significant difference in the overall abundance of *H. spumosum *between sites (hosts combined, Chow Kit = 2.0 ± 0.29; Bangsar = 0.8 ± 0.18) nor between hosts (sites combined, *R. norvegicus *= 1.2 ± 0.40; *R. rattus *= 1.8 ± 0.25). However, abundance of this nematode varied significantly between sites in *R. rattus *(higher at Chow Kit; χ^2^_1 _= 5.54, *P *= 0.019 but not in *R. norvegicus *(Table 6), although the difference between sites was in the same direction. There was no significant difference in abundance of *H. spumosum *between seasonal cycles in *R. norvegicus*, but in *R. rattus *abundance clearly increased significantly with each successive seasonal cycle (Figure 8B; χ^2^_2 _= 8.27, *P *= 0.016).

##### Hymenolepis nana

This cestode was considerably more abundant in *R. rattus *(14.2 ± 2.13) compared with *R. norvegicus *(4.8 ± 1.51; χ^2^_1 _= 11.82, *P *= 0.0006) and this pattern was consistent across both sites (Table 6). It was also a species that clearly increased in abundance in the wet season (with hosts combined χ^2^_1 _= 8.69, *P *= 0.0032). This cycle of abundance was apparent in all three seasonal cycles in *R. rattus *(χ^2^_1 _= 4.83, *P *= 0.028) with a similar pattern in cycles 1 and 2 in *R. norvegicus *but not in seasonal cycle 3 (Figure 8C).

**Figure 9:**
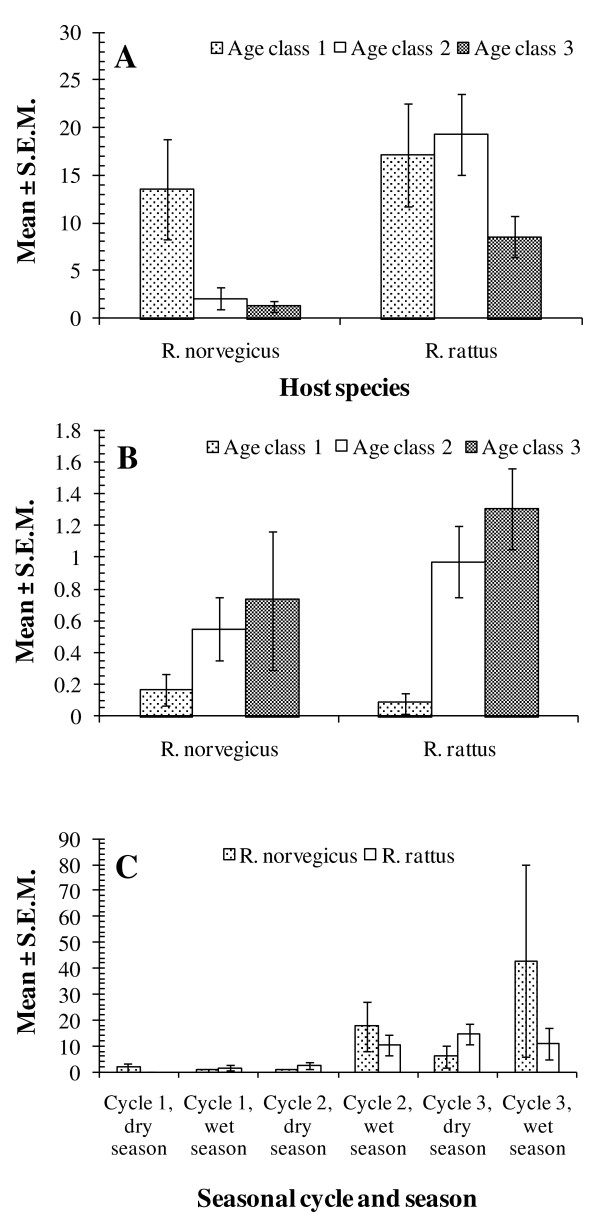
**Factors affecting the abundance of *H. nana*, *M. muris *and *N. brasiliensis***. The effect of host age on *H. nana *(A) and *M. muris *(B), and that of successive seasons in seasonal cycles on the abundance of *N. brasiliensis *(C) in each of the host species. See text for statistical analysis.

The age effect was highly significant when both hosts were combined (χ^2^_2 _= 10.22, *P *= 0.006), with declining abundance of *H. nana *as age increased. Models for *R. norvegicus *alone did not converge but analysis by non-parametric tests confirmed a significant difference between groups (Kruskal-Wallis test, *χ*^2^_2 _= 7.8, *P *= 0.02; Figure 9A). Age did not appear in the minimum sufficient model for *R. rattus*, but as with *R. norvegicus *there was a significant difference between groups when tested non-parametrically (Figure 9A; Kruskal-Wallis test, *χ*^2^_2 _= 11.3, *P *= 0.003).

##### Mastophorus muris

This species was significantly more abundant at Chow Kit (hosts combined, 1.1 ± 0.16) compared with Bangsar (0.2 ± 0.10; χ^2^_1 _= 15.32, *P *= 0.0001), and this difference between sites was consistent in both hosts (Table 6). Analysis by GLM for *R. rattus *gave *χ*^2^_1 _= 9.36, *P *= 0.002 and by non-parametric tests confirmed that there was also a significant difference between sites in *R. norvegicus *(Mann-Whitney U test, *Z *= -3.3, P = 0.001).

Overall, abundance of *M. muris *was higher in *R. rattus *(*R. rattus *= 1.0 ± 0.15, *R. norvegicus *= 0.5 ± 0.18), but this did not emerge as a significant effect in GLM, although without other factors being taken into account there was a significant difference by non-parametric analysis (Mann-Whitney *U *test *Z*= -2.36, *P *= 0.018).

There was an overall effect of host age (*χ*^2^_2 _= 12.4, *P *= 0.002) and the trend of increasing abundance with increasing age was consistent in both hosts, although only significant in *R. rattus *(Figure 9B; *χ*^2^_2 _= 14.4, *P *= 0.0008).

*M. muris *also showed significantly higher abundance in females (1.1 ± 0.18) compared with males (0.6 ± 0.14) when hosts were combined (*χ*^2^_1 _= 6.23, *P *= 0.013). Although sex did not emerge as a main effect in the GLM restricted to *R. rattus*, the non-parametric test indicated that as in the entire data-set a sex bias was apparent (*R. rattus*, males = 0.7 ± 0.18, females = 1.2 ± 0.22, Mann-Whitney *U *test *Z*= -2.2-4, *P *= 0.028). In *R. norvegicus *the mean was higher among females also, but not significantly so (females = 0.6 ± 0.31, males = 0.4 ± 0.20).

There was a significant difference in abundance between seasons (mean in wet season = 1.1 ± 0.21, and in the dry season 0.6 ± 0.13, *χ*^2^_1 _= 6.23, *P *= 0.013) when hosts were combined and this was also significant in *R. norvegicus *in which abundance was five times higher in the wet season (mean for wet season = 1.0 ± 0.43, dry season 0.2 ± 0.09, *χ*^2^_1 _= 5.37, *P *= 0.021) but not in *R. rattus *(mean for wet season = 1.2 ± 0.24, dry season 0.8 ± 0.19)

##### Nippostrongylus brasiliensis

There was no difference in abundance of *N. brasiliensis *between the hosts (*R. norvegicus *= 7.1 ± 2.77; *R. rattus *= 7.4 ± 1.50), nor between the sites when hosts were pooled (Chow Kit = 7.1 ± 1.50; Bangsar = 7.7 ± 2.77). However, this species was clearly more abundant in *R. norvegicus *at Bangsar and in *R. rattus *at Chow Kit, and this interaction was borne out by the statistical analysis (Table 6; 2-way interaction site*host species *χ*^2^_1 _= 18.81, *P *< 0.0001). No other factors or 2-way combinations were significant. However, changes in abundance of *N. brasiliensis *occurred across the seasonsal cycle and in successive seasons (Figure 9C). These data show a tendency to increasing abundance with successive cycles, particularly in *R. norvegicus *in the wet season, and an increase in abundance in *R. rattus *between seasonal cycles 1 and 3. Although, not independently significant, there was also a trend of increasing abundance with age in *R. norvegicus *(means = 1.5 ± 0.79, 4.4 ± 3.95 and 13.8 ± 6.28 for age classes 1-3, respectively) and a peak in age class 2 with subsequent decline in *R. rattus *(means = 1.2 ± 0.98, 10.5 ± 3.08 and 6.5 ± 1.74, respectively). Moreover, there was also an overall bias in the direction of male rats in both species (mean = 11.5 ± 5.03, 2.2 ± 1.49 for male and female *R. norvegicus*, and 10.0 ± 2.37, 5.5 ± 1.92 for male and female *R. rattus*, respectively), but this was not significant when other factors were included in the model.

## Discussion

Despite their importance as reservoirs of zoonotic infections, wild rats have seldom been studied thoroughly with respect to the epidemiology of their helminths, and we are not aware of any other studies in which the helminth communities of the two common commensal rat species have been compared directly by rigorous analysis with possible confounding factors taken into account. In comparison with ecological studies of wood mice and bank voles in Europe [7,9-12,35-41], there are few quantitative studies [8,42] of the helminth communities of rats exploring thoroughly a range of factors that have the potential to influence parasite burdens, and then clearly identifying and prioritising them. For the most part the literature on their helminths comprises species descriptions and lists from sites in different countries e.g. in the Middle East [43,44], Korea [45], Nigeria [46] and Malaysia [19-21,26,27]. Comparison of the helminth communities supported by each of these species was therefore one of of our key objectives.

As in many other cities around the globe, in Kuala Lumpur, *R. rattus *and *R. norvegicus *are the two dominant species of rats living commensally with people, but other species are also occasionally reported in Kuala Lumpur and the surrounding region, although seldom in high numbers [17,21,28]. The two species are closely related, with similar karyotypes [47] and *R. norvegicus *was formerly classed as a subspecies of *R. rattus *[48]. Both rat species have adapted so successfully to a commensal life style that they are universally regarded as pests. Despite the many diverse methods used to control their populations, both species are widespread globally and both support a rich community of helminths, as the current study has shown (80% were infected with at least one of the helminths identified here and the mean infracommunity species richness was 1.4), some of which are infectious to humans. In this paper we have focused on *R. rattus *and *R. norvegicus *as the obvious starting point for understanding the role of wild rats in the transmission of helminths within the city.

Our random trapping efforts yielded more than twice as many *R. rattus *compared with *R. norvegicus*, suggesting differences in the population densities of both species. Therefore, not surprisingly, the helminth species list for *R. rattus *was longer than that for *R. norvegicus*, since species richness is dependent on host density and sampling effort [49-51]. Each of the eleven species identified was recorded from *R. rattus*, whereas 3 were absent from *R. norvegicus *(*S. muris*, *G. neoplasticum *and *T. taeniaeformis*) even though each has been reported from this host in other studies [52,53]. In real terms 82% of the helminths were recovered from *R. rattus *and even when sampling effort was taken into consideration almost 63% were still from this host species. This bias in favour of *R. rattus *was also reflected at the infracommunity level in significantly higher values for species richness and for abundance of helminths, and for individual species such as *H. nana*. With all other factors taken into consideration, *R. rattus *carried significantly heavier worm burdens than *R. norvegicus *with mean values almost twice as high. In our study, therefore, and on these criteria, *R. rattus *was a more important reservoir of helminth infections than *R. norvegicus*.

We chose two quite different sites to trap rats in Kuala Lumpur. Chow Kit is the largest wet market in the city, so rats living in the site will have benefited from plentiful resources, and this site yielded most of the animals for the current project. In fact 59% of all the animals sampled were *R. rattus *trapped at this market location, so assuming that these figures reflect the overall population density, *R. rattus *clearly does well in Chow Kit and the life cycles and transmission routes of their helminths will have been well entrenched in the site. By comparison, few *R. norvegicus *were caught in Chow Kit (only 12.3% of all rats), and these accounted for only 10.9% of the helminths recovered. The smaller numbers of *R. norvegicus *here may be linked to differences in the habitat preferences of both rat species. It has been reported that *R. norvegicus *tends to favour residential sites [48], e.g. Bangsar, by occupying wall cavities and panelling of buildings, whereas *R. rattus *is typically associated with refuse and garbage tips and stores, as well as with cooked foodstuff, all readily available in the wet market of Chow Kit. Thus whereas *R. rattus *trapped at Chow Kit harboured the greatest percentage of helminths (38.9%) and showed the highest mean infracommunity species richness, *R. norvegicus *at Chow Kit had the fewest helminths and had the lowest mean species richness. Since the two rat species shared eight of the eleven species of helminths recorded in this study, it would appear that the infective stages, particularly those of the monoxenous nematodes, released from *R. rattus*, are not well transmitted to *R. norvegicus *at this site. This suggests that at least to some extent the two rats occupy different niches at Chow Kit likely to be related to their differing habitat preferences.

In the more affluent sector of the city, at our second site, Bangsar, whilst fewer *R. rattus *were caught, the *R. norvegicus *sample size was larger. Bangsar is considerably cleaner compared to Chow Kit, and garbage here is better managed and disposed of. Interestingly, here the two rat species accounted for very similar percentages of the helminths recovered. Because at Chow Kit *R. rattus *accounted for a large proportion of the helminths and *R. norvegicus *for relatively fewer, when data for the rat species were pooled, and expressed in percentage terms there was no real difference in helminths recovered from each of the two sites. The key difference was that in Chow Kit *R. rattus *was clearly more important than *R. norvegicus *as a carrier of helminths. Thus the disparity between the host species was essentially site specific and not equally evident at Bangsar.

Of the eleven species of helminths recorded, none of the nematodes are generally considered to be human infectious, although some authorities consider some to have zoonotic potential [28]. In addition to those we found, rats can be expected to harbour the infective stages of *Toxocara cati *and *T. malaysiensis *[54,55], both of which are known to exist in the local cat population (JW Lewis and SN Mohd Zain, unpublished observations), but in the current work the musculature of the rats was not examined in sufficient detail to reveal these parasites. There was no evidence in the current study of *Calodium hepaticum *(previously known as *Capillaria hepatica*, [56]), in the liver of the rats, although three of 50 rats examined earlier were infected with this parasite and it is known to have potential to cause human infections [28]. Only two of the cestodes have been reported to infect humans more than just on the rare incidental occasion. *H. diminuta *has occasionally been reported from humans [57-59], and its occurrence in human hosts is linked with the accidental consumption in food of flour beetles, which act as intermediate hosts. There is, however, controversy as to whether the *H. nana *in rodents is the same or a different species to that infecting humans, although recent data support the lack of infectivity of human isolates in rodents [60,61]. The dwarf tapeworm of humans can be common in some human communities [62-65] and it has been recorded in schoolchildren in Kuala Lumpur and other regions of Malaysia in the past [66-68]. Likewise, *H. diminuta *has also been reported from human populations in Malaysia [68], although to our knowledge not from Kuala Lumpur. Given that over a quarter of each of the rat species in both sites were infected with *H. diminuta*, (and as high as 57.5% of *R. norvegicus *in Bangsar) and similar numbers carried *H. nana*, this must represent a public health risk, especially in the latter case where the life cycle can be direct without involvement of insect intermediate hosts, and therefore contamination of food for human consumption at market and street stalls is a high probability in both sites.

In spite of the high prevalence of *H. diminuta *in *R. norvegicus *in Bangsar, most cestodes were recovered from *R. rattus*, even relatively when sampling effort was taken into account, and this was largely due to the higher abundance of *H. nana *in this host. *H. nana *was the dominant species in 3 of the 4 data-subsets, and the abundance of this cestode in *R. rattus *was almost 3 times as high as that in *R. norvegicus*. The dominance of cestodes in this study does have some precedents in tropical countries. For example, it has been reported that *H. diminuta *was the only helminth recovered from an urban rat population in Qatar [42], but this may have been a consequence of the harsh arid environment in the outskirts of Doha reducing helminth richness and diversity.

Interestingly, nematodes showed a different preference for rat hosts in each of the two sites. Thirty eight percent of all nematodes were recovered from *R. norvegicus *at Bangsar, whilst 47.2% were from *R. rattus *at Chow Kit, and to a large extent these values were driven by the abundance of *N. brasiliensis*. At Bangsar this species was 12 times more abundant in *R. norvegicus *and in Chow-Kit over 100 times more abundant in *R. rattus*. This again strongly suggests that whilst both species of rats were recovered from both sites, their exact ecological niches did not greatly overlap. *R. norvegicus *generally displace the black rat in areas where humans live. In addition to being larger and more aggressive, the change from wooden structures and thatched roofs, typical of poorer quarters of the city, to bricked and the tiled buildings found at Bangsar, favours the burrowing brown rats over the more arboreal black rats. Moreover, brown rats eat a wider variety of foods, and are more resistant to weather extremes. *N. brasiliensis *is transmitted by infective motile skin penetrating L3 larvae which contaminate the nests and runs that *R. rattus *uses in territories that are more likely to include refuse tips which provide optimum conditions for the survival and hatching of eggs of *N. brasiliensis*. In the cleaner environment of Bangsar, *R. norvegicus *may live in contaminated burrows, while *R. rattus *may avoid comparable levels of exposure by the absence of freely available refuse and its arboreal habits.

The climate in Kuala Lumpur is warm throughout the year with no markedly contrasting seasons comparable to those experienced in temperate climatic zones, but in general there is more rain between October to February and this we have treated as the wet season. Although our analysis detected seasonal effects in some cases, few were independent of interactions with other factors, clearly evident in the summary data and consistent across the three seasonal cycles. One of the exceptions was *M. muris *which showed a higher prevalence in *R. rattus *in all three wet seasons compared to the preceding dry seasons, although a dip in prevalence after a rainy season was not always evident, as for example in the period between cycle 1 and 2. Abundance was also higher in the wet season in the combined data-set and in *R. norvegicus*. Whilst not significant, nor independent of other factors in the case of *R. norvegicus*, a similar pattern of prevalence was evident in cycles 1 and 2 but not 3 (data not shown earlier but in cycle 3 prevalence was very low with only one of the 40 rats caught in the combined dry and wet seasons being infected). Rodents become infected with *M. muris *when they feed on infected intermediate hosts (Orthoptera, Dermaptera and Dictyoptera [69] and even Siphonaptera [70]), that harbour the infective L3 stages, and it is conceivable that this seasonal pattern of occurrence in rats was to some extent determined by the availabilty of arthropod hosts and their annual population cycles. A striking seasonal pattern with highest prevalence and abundance of this species in *R. rattus *was recorded in the early autumn and winter periods on North Island of New Zealand, at a season that also coincided with the wetter part of the year [71]. The closely related species *M. dipodomis *showed a higher prevalence in the late summer/autumn period in *Dipodomys *spp. and *Perognathus *spp. in New Mexico, USA [72]. However, seasonal differences in prevalence and abundance of other spirurid nematodes have pointed to higher transmission in the summer months (e.g. *Pterygodermatites peromysci *in *Peromyscus leucopus *[13]).

A similar pattern relating to abundance was found for the tapeworm *H. nana*, with mean abundance being consistently higher in the wet season in *R. rattus*, and in the first two seasonal cycles in *R. norvegicus*. This species can exploit intermediate hosts such as the tenebrionid beetles but it can also be transmitted directly, since its eggs can hatch in the intestine of rodents and complete the cysticeroid stage in the small intestine as well as the subsequent maturation to fecund adults. The seasonal pattern, however, does suggest that transmission may have been dependent on external sources, but whether egg survival in the dry season was poorer or whether suitable insect hosts were scarce is not known.

The significant seasonal effects detected in *H. nana *and *M. muris*, largely explained the patterns observed in other parameters used to asses helminth communities when data from all helminths were combined. For example species richness was higher in the wet compared to dry seasons, although in the case of *R. rattus*, the lack of this effect in cycle 3, led to the loss of the overall seasonal variation when data were combined across 3 cycles. Mean abundance of helminths also increased in the wet season in both hosts, but additionally there was a steady progression of increasing abundance across cycles 1, 2 and 3, suggesting longer-term trends and possibly cycles over a longer time frame than that detectable in a 3 year investigation.

Longer term patterns were evident through the significant differences between cycles, but again in only a few cases did they form clear unidirectional trends over time. Nevertheless, there was a significant trend of increasing helminth diversity in *R. rattus *but not in *R. norvegicus*, suggesting that helminth community structure in the former was changing over time. At the individual species level, the abundance of *H. spumosum *increased with each successive cycle in *R. rattus *but not in *R. norvegicus*, whilst that of *N. brasiliensis *increased in both hosts. Both of these nematodes are monoxenous and therefore do not depend on intermediate hosts, and transmission is by eggs and infective L3 larvae, respectively.

Conversely, there were also some interesting downward trends with time. For example the prevalence of *H. diminuta *drifted downwards with successive cycles in both rat hosts in Bangsar, but not in Chow Kit, suggesting that the transmission was decreasing with time and that the management policy for Bangsar, which is always kept clean, was paying off in terms of disease transmission. With markets combined, abundance also showed a clear decrease with time in *R. rattus *but not in *R. norvegicus*. However, the abundance of *H. nana *fell with successive cycles in *R. norv*egicus but not in *R. rattus*, in which abundance was stable in each of the wet seasons and there was an upward trend with successive dry seasons. Whether these patterns actually represent dynamic changes resulting from changes in the risk of transmission locally or whether they are constituents of longer-term cycles, in both cases driven by biological/ecological factors, or just spurious variation between years is not yet clear, and will only become more apparent when data collection is continued over an even longer period of time.

Helminths are renowned for their chronic long-lasting infections and the poor protective responses that they elicit in their hosts [73,74], and not surprisngly they are often found to accumulate with host age [75]. In our analysis, examples included the increase in helminth diversity and increasing species richness with age in *R. rattus *, increasing prevalence of *H. spumosum *with age in both rats species, increasing abundance of *N. brasiliensis *with age in *R. norvegicus *and increasing prevalence and abundance of *M. muris *in both species of rats. The strongest independent effects of age were observed in the last named species, a stomach dwelling spirurid that is relatively large and robust with a thick cuticle [76]. It takes over 5 weeks to begin laying eggs and is clearly well adapted for long term survival in its host [69].

One species that is known to stimulate potent immunity in its host is *H. nana *[77], and our data were consistent with this concept in so far as prevalence and abundance declined with host age in both host species, suggesting that after exposure in early life rats became immune to further infection and rejected their earlier acquired worm burdens. There was also some indication that the abundance of *N. brasiliensis *declined in the oldest *R. rattus*, but not in *R. norvegicus*, and this is another species that is known to elicit potent protective immunity in its host [78,79].

Differences between the host sexes in helminth burdens are relatively infrequently observed in naturally infected rodent populations [80]. Here, while we did not find any strong independent effects of host sex on prevalence of any of the species that were examined, there were significant differences between the sexes in the overall abundance of helminths. When all helminth species were pooled, male rats harboured more worms than females, but at the individual species level only two showed significant sex effects and in both they were in the opposite direction. Thus female *R. rattus *harboured more *H. diminuta *than males, and more *M. muris*. Where host sex bias has been detected in helminth infections, it has been more often in favour of males which are considered to have weaker immune responses because of the immunodepressive effects of male sex hormones such as testosterone [81-83]. However, female host bias in *M. muris *has been observed previously [52], although not in all studies [72,84] and in some a bias in the opposite direction has been reported also [71].

## Conclusions

While assessing the role of the two common commensal rat species as reservoirs of helminth infections, we have also explored the role of other intrinsic and extrinsic factors, and in doing so, we were able to take their influence into consideration when interpreting possible differences between the rat hosts. Although *R. rattus *were more common in the present study and represented a greater proportion of the total sample of rats that were analyzed and accounted for most of the helminths, both in terms of species and quantitatively in terms of actual numbers of worms, infections in these hosts were dynamic with significant seasonal and year effects, but nevertheless largely concentrated in rats from the wet market at Chow Kit, where the majority were trapped. In contrast helminth infections in *R. norvegi*cus were more stable, less affected by site, although where there was a bias it was in favour of the more affluent site at Bangsar. Both of these two species are commensal urban dwellers, but the parasitological evidence from this study, based on their contrasting patterns of infection, indicates that whilst they share the majority of helminth species that are endemic in the region, they most likely occupy different niches in Kuala Lumpur with limited physical overlap and opportunity for cross infection. We conclude also that nematodes are of little if any significance to the human population, but the two cestode species *H. diminuta *and *H. nana *do represent a health risk, and hence further epidemiological investigations of the parasites of rat populations in other urban sites in Malaysia are required.

## Competing interests

The authors declare that they have no competing interests.

## Authors' contributions

SNMZ and JWL conceived the study. SNMZ collected the data. JMB analysed the data and all three authors wrote the manuscript and approved the final version.
